# Biomechanical analysis of smart walking shoe sending movement information to display device by radio communication

**DOI:** 10.1186/1757-1146-7-S1-A121

**Published:** 2014-04-08

**Authors:** Seung-Bum Park, Kyung-Deuk Lee, Dae-Woong Kim, Jung-Hyeon Yoo, Kyung-Hun Kim

**Affiliations:** 1 Footwear Biomechanics Team, Footwear Industrial Promotion Center, Busan, Korea

## 

The purpose of this study was to find the difference in foot pressure patterns when wearing smart walking shoes. Foot pressure measurement is an established tool for the evaluation of foot function [1]. These measurements assess the effect of structural changes, which may occur as a complication of pathologies such as diabetes, and therefore have been suggested as one of the key tools in ulcer risk estimation [2].

The subjects who took part in the test consist of 5 elderly people and 5 young people. The physical features of the elderly people that were recruited for the study are shown below: 5 healthy male subjects (elderly people) with an average age of 62.0 yrs (S.D 1.0 yrs), weight of 69.4 kg (S.D 10.0 kg), height of 168.8 cm (S.D 5.3 cm) and a foot size of 270.0 mm (S.D 0.0 mm). 5 healthy male subjects (young people) with an average age of 27.2 yrs (S.D 4.1 yrs), weight of 75.2 kg (S.D 4.6 kg), height of 175.4 cm (S.D 4.0 cm) and a foot size of 270.0 mm (S.D 0.0 mm). Ten males (5 elderly people, 5 young people) walked on a treadmill wearing three different shoes. Foot pressure data (Contact areas, Maximum forece, Peak pressure, Maximum mean pressure) was collected using a Pedar-X mobile system (Novel Gmbh., Germany) operating at the 1,000 Hz.

**Figure 1:**
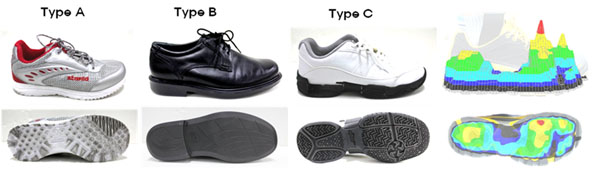
Type A: development shoes, Type B: control shoes, Type C: smart walking shoes

**Table 1:** Result of Foot Pressure

Subjects	Mask	Contact area (cm^2^)			Maximum force (N)		
		
		A	B	C	A	B	C
Young	Total	142.877±6.584	131.852±10.934	142.342±5.754	711.105±59.923	740.921±95.996	701.841±60.198
	M1	68.663±1.716	63.023±5.373	68.629±0.584	621.023±89.605	606.018±168.64	601.982±86.053
	M2	33.443±5.540	29.811±4.185	33.331±5.175	133.911±8.162	113.943±21.044	140.778±14.482
	M3	40.770±0.000	39.019±2.200	40.383±0.753	468.385±42.442	500.382±46.850	471.992±27.290

Elderly	Total	139.403±2.996	128.966±5.757	138.099±4.256	592.178±95.362	605.047±81.495	596.161±100.23
	M1	68.119±3.213	64.589±5.796	68.221±3.705	526.524±75.498	545.776±74.082	546.801±90.669
	M2	30.514±2.751	24.877±5.708	29.140±4.599	110.238±25.983	78.007±31.900	96.843±29.870
	M3	40.770±0.000	39.503±1.290	40.736±0.060	386.392±94.017	428.618±84.020	397.017±94.609

**Subjects**	**Mask**	**Peak pressure (kPa)**			**Maximum mean pressure (kPa)**		
		
		**A**	**B**	**C**	**A**	**B**	**C**

Young	Total	270.869±70.830	264.823±50.235	247.067±50.477	86.504±3.965	99.139±8.358	88.268±7.415
	M1	258.458±83.422	243.390±75.894	235.239±59.953	94.519±9.360	101.522±19.698	93.791±10.480
	M2	84.522±14.058	87.059±19.501	88.965±22.004	46.799±7.466	48.141±11.532	47.585±8.937
	M3	184.082±25.588	213.283±16.517	190.809±25.685	115.573±11.070	130.126±11.114	119.693±10.816

Elderly	Total	189.973±27.832	213.509±21.026	213.564±45.475	76.358±3.203	85.410±3.122	77.770±7.078
	M1	188.168±27.811	212.000±20.270	213.564±45.475	81.126±5.774	87.280±3.075	82.372±8.326
	M2	66.064±6.977	67.977±18.067	57.432±9.937	39.860±6.977	38.683±7.441	36.246±6.136
	M3	134.086±33.163	165.232±33.123	140.901±30.023	94.773±23.062	110.738±22.902	97.650±23.442

The results are as follows:

1. Young people

In comparison with the Type B (control shoes):

1) Type A (development shoes)

a)The contact area of foot (Total) by increased 8.36%, forefoot (M1) by increased 8.95%, midfoot (M2) by increased 12.18% and rearfoot (M3) by increased 4.48%. b)The maximum force of foot (Total) by decreased 4.02%, rearfoot (M3) by decreased 6.39%, while the maximum force of forefoot (M1) by increased 2.48% and midfoot (M2) by increased 17.52%. c)The peak pressure of foot (Total) by increased 2.28%, forefoot (M1) by increased 6.19%, while the peak pressure of midfoot (M2) by decreased 2.91% and rearfoot (M3) by decreased 13.69%. d)The maximum mean pressure of foot (Total) by decreased 12.74%, forefoot (M1) by decreased 6.90%, midfoot (M2) by decreased 2.79% and rearfoot (M3) by decreased 11.18%.

2) Type C (smart walking shoes)

a)The contact area of foot (Total) by increased 7.96%, forefoot (M1) by increased 8.90%, midfoot (M2) by increased 11.81% and rearfoot (M3) by increased 3.50%. b)The maximum force of foot (Total) by decreased 5.27%, forefoot (M1) by decreased 0.67% and rearfoot (M3) by decreased 5.67%, while the maximum force of midfoot (M2) by increased 23.55%. c)The peak pressure of foot (Total) by decreased 6.70%, forefoot (M1) by decreased 3.35% and rearfoot (M3) by decreased 10.54%, while the peak pressure of midfoot (M2) by increased 2.19%. d)The maximum mean pressure of foot (Total) by decreased 10.97%, forefoot (M1) by decreased 7.62%, midfoot (M2) by decreased 1.15% and rearfoot (M3) by decreased 8.02%.

2. Elderly people

In comparison with the Type B (control shoes):

1) Type A (development shoes)

a)The contact area of foot (Total) by increased 8.09%, forefoot (M1) by increased 5.47%, midfoot (M2) by increased 22.66% and rearfoot (M3) by increased 3.21%. b)The maximum force of foot (Total) by decreased 2.13%, forefoot (M1) by decreased 3.53% and rearfoot (M3) by decreased 9.85%, while the maximum force of midfoot (M2) by increased 41.32%. c)The peak pressure of foot (Total) by decreased 11.02%, forefoot (M1) by decreased 11.24%, midfoot (M2) by decreased 2.81% and rearfoot (M3) by decreased 18.85%. d)The maximum mean pressure force of foot (Total) by decreased 10.60%, forefoot (M1) by decreased 7.05% and rearfoot (M3) by decreased 14.42%, while the maximum force of midfoot (M2) by increased 3.04%.

2) Type C (smart walking shoes)

a)The contact area of foot (Total) by increased 7.08%, forefoot (M1) by increased 5.62%, midfoot (M2) by increased 17.14% and rearfoot (M3) by increased 3.12%. b)The maximum force of foot (Total) by decreased 1.47%, rearfoot (M3) by decreased 7.37%, while the maximum force of forefoot (M1) by increased 0.19% and midfoot (M2) by increased 24.15%. c)The peak pressure of foot (Total) by increased 0.03%, forefoot (M1) by increased 0.74%, while the peak pressure of midfoot (M2) by decreased 15.51% and rearfoot (M3) by decreased 14.73%. d)The maximum mean pressure of foot (Total) by decreased 8.95%, forefoot (M1) by decreased 5.62%, midfoot (M2) by decreased 6.30% and rearfoot (M3) by decreased 11.82%.

As a result of analysis, it has been found that Type A and Type C have lower foot pressure (Total, M3) than Type B. Also, Type A and Type C show superior performance compared to Type B in all mask at contact area. Type A and Type C shoes will be used to reduce foot pressure and increase comfort and fitting.
